# Tomato expressing *Arabidopsis* glutaredoxin gene *AtGRXS17* confers tolerance to chilling stress via modulating cold responsive components

**DOI:** 10.1038/hortres.2015.51

**Published:** 2015-11-11

**Authors:** Ying Hu, Qingyu Wu, Stuart A Sprague, Jungeun Park, Myungmin Oh, C B Rajashekar, Hisashi Koiwa, Paul A Nakata, Ninghui Cheng, Kendal D Hirschi, Frank F White, Sunghun Park

**Affiliations:** 1 Department of Horticulture, Forestry, and Recreation Resources, Kansas State University, Manhattan, KS 66506, USA; 2 Department of Horticultural Science, Texas A&M University, College Station, TX 77843, USA; 3 United States Department of Agriculture/Agricultural Research Service, Children’s Nutrition Research Center, Department of Pediatrics, Baylor College of Medicine, Houston, TX 77030, USA; 4 Department of Plant Pathology, Kansas State University, Manhattan, KS 66506, USA

## Abstract

Chilling stress is a production constraint of tomato, a tropical origin, chilling-sensitive horticultural crop. The development of chilling tolerant tomato thus has significant potential to impact tomato production. Glutaredoxins (GRXs) are ubiquitous oxidoreductases, which utilize the reducing power of glutathione to reduce disulfide bonds of substrate proteins and maintain cellular redox homeostasis. Here, we report that tomato expressing *Arabidopsis* GRX gene *AtGRXS17* conferred tolerance to chilling stress without adverse effects on growth and development. *AtGRXS17*-expressing tomato plants displayed lower ion leakage, higher maximal photochemical efficiency of photosystem II (Fv/Fm) and increased accumulation of soluble sugar compared with wild-type plants after the chilling stress challenge. Furthermore, chilling tolerance was correlated with increased antioxidant enzyme activities and reduced H_2_O_2_ accumulation. At the same time, temporal expression patterns of the endogenous C-repeat/DRE-binding factor 1 (*SlCBF1*) and *CBF* mediated-cold regulated genes were not altered in *AtGRXS17*-expressing plants when compared with wild-type plants, and proline concentrations remained unchanged relative to wild-type plants under chilling stress. Green fluorescent protein -AtGRXS17 fusion proteins, which were initially localized in the cytoplasm, migrated into the nucleus during chilling stress, reflecting a possible role of AtGRXS17 in nuclear signaling of chilling stress responses. Together, our findings demonstrate that genetically engineered tomato plants expressing *AtGRXS17* can enhance chilling tolerance and suggest a genetic engineering strategy to improve chilling tolerance without yield penalty across different crop species.

## Introduction

Chilling stress, here defined as exposure to temperatures ranging from 0 °C to 12 °C, adversely affects the growth and development of many crop species of tropical or subtropical origin, limiting agricultural productivity.^1^ Tomato (*Solanum lycopersicum*) is also subject to chilling stress due to extensive cultivation in temperate regions despite a tropical origin. Germination, vegetative growth, flowering, fruit set development, ripening, and postharvest are all affected by chilling stress.^2^ In the past two decades, while numerous traditional or molecular breeding efforts have been undertaken to improve tomato chilling tolerance, significant successful progress has not been made due to the complexity of chilling tolerance traits, linkage drag that affects yield and quality, and the lack of quantifiable physiological parameters related to chilling tolerance.^3,4^ Thus, genetic engineering should be considered as an alternative approach to improve chilling tolerance in tomato.

One of the well utilized gene families to genetically engineer chilling tolerance in tomato is C-repeat-binding factor/dehydration responsive element-binding protein 1 (*CBF/DREB1*) (ref. 5). Plants that are adapted to chilling conditions, including *Arabidopsis*, sense low temperatures and activate expression of members of the *CBF/DREB1* gene family of transcription factors, which includes *CBF1*, *CBF2*, and *CBF3.* The protein products of the genes, in turn, regulate the expression of cold-regulated(*COR*) genes.^5^ *COR* genes constitute the *CBF* regulon, which includes more than 100 genes, and have been shown to be essential for both chilling tolerance and cold acclimation in plants.^3,4^ Although tomato has a CBF-mediated cold-responsive pathway, the tomato *CBF* regulon consists of fewer and less functionally diverse genes than that of *Arabidopsis.*^6,7^ Therefore, ectopic expression of genes involved in cold responsive pathways of *Arabidopsis*, particularly the *CBFs*, may have a different response and adaptation to cold stress than in tomato.^6,7^ Moreover, growth retardation has been attributed to ectopic expression of *CBF* genes in tomato plants, further limiting the effectiveness of ectopic expression of *CBFs* for engineering chilling tolerant tomato.^7,8^ Similarly, constitutive expression of *CBFs* in *Arabidopsis*,^9^ potato,^10^ and *Brassica napus*^11^ enhances freezing tolerance but induces dwarfism, in part, due to the accumulation of DELLA proteins, a family of nuclear growth-repressing regulatory proteins.^12^

An inevitable consequence of chilling stress is the accumulation of reactive oxygen species (ROS), which is one of the major factors leading to cold injury.^1,13^ Although ROS can act as signal molecules for stress responses, excess ROS causes oxidative damage to various cellular components including membrane lipids, structural proteins, and enzymes, and leads to inhibition of plant growth and development.^13–15^ Therefore, ROS levels must be regulated in plants through the coordination of ROS production and scavenging to manage oxidative damage while maintaining ROS-mediated signaling.^16,17^

Glutaredoxins (GRXs) are small ubiquitous oxidoreductases of the thioredoxin (Trx) family and catalyze reversible reduction of disulfide bonds of substrate proteins by using the reducing power of glutathione (GSH) (ref. 17). The family members are present in both prokaryotes and eukaryotes and are necessary for redox buffering, heavy metal detoxification, plant development, plant–pathogen interactions, iron homeostasis, and oxidative stress response.^17–21^ In addition, previous studies suggest that individual GRX family members may have multiple functions in plants. For instance, PvGRX5 from *Pteris vittata* plays roles in both arsenic and heat stress tolerance.^22,23^ AtGRXS13 from *Arabidopsis* functions in both pathogen and photo-oxidative stress responses,^24,25^ and AtGRXS17 has critical functions in regulating cellular ROS metabolism,^26^ shoot apical meristem development,^27^ and heat stress tolerance.^28^ Yet, the function of AtGRXS17 in oxidative damage due to chilling stress of plants, how AtGRXS17 affects chilling stress responses, chilling-associated gene expression, plant growth and development, and signaling under chilling stress, is unknown.

In this study, we ectopically expressed *AtGRXS17* in tomatoes to investigate the role of *AtGRXS17* in chilling tolerance. Further, we generated the stress inducible *RD29A*::*AtCBF3*-expressing tomatoes as positive control lines and compared chilling tolerance of the plants with *AtGRXS17*-expressing and wild-type lines. The cellular localization of AtGRXS17 under chilling stress conditions was also characterized by using transient expression in *Nicotiana tabacum*. Temporal expression patterns of the endogenous *SlCBF1* and *CBF-* mediated-*COR* genes as well as physiological and biochemical responses were analyzed to investigate whether ectopic expression of *AtGRXS17* enhanced chilling tolerance through a CBF-independent manner during chilling stress. This work provides an innovative perspective on engineering chilling tolerance in tomato.

## Materials and methods

### Bacterial strain, plasmid, and tomato transformation

*AtGRXS17* coding region was cloned into pBICaMV vector driven by the cauliflower mosaic virus (CaMV) *35S* promoter as described previously.^28^ The *AtCBF3* coding region was also cloned into pMDC99 vector driven by a cold-inducible *RD29A* promoter^29^ to avoid any negative effects on plant growth due to constitutive expression of *CBFs* (ref. 9). Binary plasmids pMDC-AtCBF3 and pBICaMV-AtGRXS17 were introduced into *Agrobacterium tumefaciens* strain LBA4404 using the freeze-thaw method^30^ and used for generating stable transgenic lines, respectively. Seeds of tomato *S. lycopersicum* L. (cv Rubion) were surface sterilized and germinated on the Murashige and Skoog inorganic salt medium,^31^ and tomato transformation was performed via *Agrobacterium*-mediated transformation method using cotyledon and hypocotyls explants as described.^32^

### Growth condition and tolerance analysis of tomato

T2 generation of *AtGRXS17*-, *AtCBF3*-expressing or wild-type tomato seeds were surface-sterilized, germinated, and grown on pots containing Metro Mix (900) growing medium in growth chamber. The temperature of the growth chamber was maintained at 24 °C/20 °C (day/night) under a 16-h photoperiod, and the light intensity was maintained at 300 μmol photons m^−2^ s^−1^. The plants were regularly watered and fertilized on a weekly basis with 20:20:20 fertilizer (Scotts). For the chilling treatment, 4-week-old *AtGRXS17*-, *AtCBF3*-expressing or wild-type seedlings were treated at 4 °C (day/night) for 3 weeks in a walk-in growth chamber, and then recovered in normal growth conditions as mentioned above for 2 weeks. The electrolyte leakage and chlorophyll fluorescence were tracked during the first 7-day chilling treatment.

For oxidative stress treatment, 7-day-old *AtGRXS17*-expressing and wild-type seedlings grown on the MS media were transferred into the MS medium with or without 20 μM methyl viologen (MV) in magenta boxes and incubated for 14 days. The primary root length was measured after harvest.

### RNA extraction and qRT-PCR

Total RNA was isolated using the Qiagen Plant RNeasy Kit from leaves of tomato plants according to the manufacturer’s instructions. The complementary DNA (cDNA) was synthesized using the iScript Select cDNA synthesis kit (Bio-Rad, Hercules, CA, USA). One microliter of the reverse transcription reaction solution was used as a template in a 25 μL PCR solution. Real-time qRT-PCR was performed in 25 μL reactions contain 10.5 μL cDNA, 1 μL 10 mM of each primer, and 12.5 μL SYBR Green PCR Master Mix (Bio-Rad). Analysis was performed using the Bio-Rad IQ3 (Bio-Rad). Primer efficiencies were measured and relative expression level was calculated using the comparative *C_t_* method.^28^ *SlPP2ACS* was used as a normalization control.^33^ The primers for PCR were listed in the supplementary data (Supplementary Table S1).

### Electrolyte leakage and Fv/Fm ratio

Injury to plants was characterized by measuring chlorophyll fluorescence and electrolyte leakage of leaves as described previously.^34^ Chlorophyll fluorescence from the adaxial side of the leaf was monitored using a portable chlorophyll fluorometer (PEA, Hansatech Instruments, Ltd., King's Lynn, England, UK). Photochemical efficiency of leaves as determined by chlorophyll fluorescence ratios (Fv/Fm) was monitored during and after the chilling treatment. Measurements were made during the light cycle on the leaves using the saturation pulse method after 30 min of dark adaption. For electrolyte leakage, tomato leaf samples were incubated in 15 mL of distilled water for 10 h to measure the initial electrolyte leakage using a YSI conductance meter (Model 32, YSI Inc., Yellow Springs, OH, USA). The samples were subjected to 80 °C for 2 h to release the total electrolytes and then held at room temperature for 10 h. The final conductivity on the leachate was measured to determine the percent electrolyte leakage from the leaf samples.

### Histochemical detection of H_2_O_2_

H_2_O_2_ was visually detected *in situ* in the leaves of tomato plants by staining with 3,3′-diaminobenzidine (DAB) as described previously^35,36^ with modification. Briefly, the terminal leaflet of the first fully expanded leaf was sampled from wild-type and *AtGRXS17*-expressing 4-week-old plants. Leaflets were completely immersed with the DAB solution (1 mg mL^−1^, pH 3.8, added with 0.05% (v/v) Tween 20 and 5% (v/v) 200 mM Na_2_HPO_4_). The sampled leaves were placed in petri dishes covered with aluminum foil under 4 °C until brown precipitate was observed (∼3 days) and then cleared in boiling ethanol (96%) for 10 min. H_2_O_2_ accumulation was detected as brown spots after DAB staining. Quantitative analyses of DAB staining were performed using image J analysis.^28^

### Enzyme assays

Superoxide dismutase (SOD) was measured using a modified nitro blue tetrazolium (NBT) method.^37^ Reactions were set up containing 50 mM phosphate buffer (pH 7.8), 13 mM methionine, 75 μM p-nitro blue tetrazolium, 2 μM riboflavin, 10 μM EDTA, and 50 μL of enzyme extract. The reaction was initiated by illuminating the samples under a 15 W fluorescent tube. Four samples containing pure buffer instead of extract were also made. Two of these were placed in a dark place and used as the blank and another two samples were placed under the 15 W fluorescent tubes as the control. Both controls and those containing enzyme extract were placed under lights for 10 min. The absorbance of the samples at 560 nm was measured, and one unit of SOD activity was defined as the amount of enzyme that inhibits the NBT photoreduction by 50%. The specific activity of SOD was expressed as U mg^−1^ protein.

Catalase (CAT) activity was measured by using the Amplex Red Catalase Assay Kit (Molecular Probes, Eugene, OR, USA). In brief, initially reaction mixtures containing 25 μL CAT-containing samples and 25μL 40 μM H_2_O_2_ were incubated at room temperature for 30 min. Then 50 μL 100 μM Amplex Red reagent containing 0.4 U mL^−1^ horseradish peroxidase was added to each microplate well containing the samples and controls and incubated at 37 °C for 30 min under protected from light. CAT activity was determined by measuring the absorbance at 560 nm using a microplate reader. One unit was defined as the amount of enzyme that will decompose 1.0 μM of H_2_O_2_ per minute at pH 7.0 at 25 °C. The specific activity of CAT was expressed as mU μg^−1^ protein.

Guaiacol peroxidase (POD) activity was measured by a modified method of Maehly and Chance.^38^ The reaction was set up containing 50 mM sodium acetate buffer pH 5.6, 0.2% guaiacol, 0.3% H_2_O_2,_ and enzyme extract. The increase in absorbance due to the oxidation of guaiacol to tetraguaiacol was monitored at 470 nm. One unit was defined as 0.01 absorbance increase per minute at 470 nm. The specific activity of POD was expressed as unit mg^−1^ protein.

### Proline determination

The proline content was determined with colorimetric assay as described previously.^39^ Four-week-old plants were treated at 4 °C and the terminal leaflet of the first fully expanded leaf was sampled from wild-type and *AtGRXS17*-expressing plants were harvested on 0, 1, 3, 5, 7, 12, and 21 days, respectively. One hundred milligram of leaf tissue was homogenized in liquid nitrogen and suspended with 500 μL 3% sulfosalicylic acid (5 μL mg^−1^ fresh weight (FW)). The obtained extraction was centrifuged for 5 min at room temperature with maximum speed. Then 100 μL supernatant of the plant extract to 500 μL reaction mixture (100 μL of 3% sulfosalicylic acid, 200 μL glacial acetic acid, 200 μL acidic ninhydrin) was added. The tubes were incubated at 96 °C for 60 min and the reaction was terminated on ice. To extract the samples with toluene, 1 mL toluene to the reaction mixture was added, and the samples were vortexed for 20 s and left on the bench for 5 min to allow the separation of the organic and water phases. The upper organic phase was used for measurement of proline. The abosorbance was determined at 520 nm using toluene as reference. The proline concentration was determined using a standard concentration curve and calculated on FWbasis (mg g^−1^).

### Total soluble sugar content determination

The soluble sugar content was determined by anthrone method as described previously.^40^ Total soluble sugar was measured by anthrone reagent. Leaf samples (100–400 mg) were homogenized in liquid nitrogen and suspended with 10 mL deionized water. Samples were placed in a water bath for 45 min at 70 °C, vortexed thoroughly every 15 min and the reaction was terminated on ice. Samples were centrifuged at 3000 rpm for 10 min and then diluted 10 times. Five milliliter of anthrone solution (100 mg anthrone/50 mL sulfuric acid) was added into 2.5 mL diluted supernatant solution. After vortexing thoroughly, samples were placed in a water bath for exactly 10 min at 100 °C and the reaction was terminated on ice. The absorbance was determined at 630 nm using deionized water as a reference. The total soluble sugar concentration was determined using a standard concentration curve and calculated on FW basis (mg mg^−1^).

### Time-course analysis of stress-responsive genes

To evaluate the effects of AtGRXS17 on the expression of *dehyrin Ci7*, *dehydrin-lik*e, *proteinase inhibitor*, *glycine rich, SlCBF1, SlCAT1, SlSOD, SlFESOD, SlTPX1*, and *SlTPX2* genes, chilling treatments were applied to 4-week-old *AtGRXS17*-expressing and wild-type plants. For chilling treatment, the plants grown in soil pots were moved to a growth chamber set at 4 °C. Leaves from wild-type and *AtGRXS17*-expressing tomato plants were harvested and measured after being treated for 0, 4, 8, 24, and 48 h, respectively.

### Subcellular localization of AtGRXS17 in plant cells

To investigate the subcellular localization of AtGRXS17 in plant cells under chilling stress, an *Agrobacterium*-mediated transient expression assay was conducted in tobacco leaves (*N. tabacum*) as described previously.^41^ Full-length *AtGRXS17* was fused to the C-terminus of green fluorescent protein (GFP) using a procedure described previously.^42^ The *GFP-AtGRXS17* construct was made by LR reaction (Invitrogen, Carlsbad, CA, USA) between the binary vector pB7WGF2 (ref. 43) and the entry vector carrying *AtGRXS17* (pENTER-4, Invitrogen). pB7WGF2::GFP-AtGRXS17 was introduced into *A. tumefaciens* LBA4404. A modified green fluorescent protein construct (Free GFP construct) was made by the Cre-loxP recombination system using a procedure described previously.^44^ pSK001 construct was generated by inserting a 1.9 kb SacI-HindIII fragment from pBV579 (containing 35S::mCherry::NLS::Tnos) into the unique SacI and HindIII sites of pCAMBIA1300. These three constructs were transformed into *A. tumefaciens* LBA4404. *A*. *tumefaciens* cells were cultivated overnight, and 5 mL of the culture was pelleted and resuspended with infiltration medium (250 mg D-glucose, 5 mL MES stock solution, 5 mL Na_3_PO_4_•12H_2_O stock solution, 5 mL 1 M acetosyringone stock solution; make up to 50 mL with ddH_2_O.) to 0.1 optical density. *A. tumefaciens* cells were infiltrated into tobacco leaves, and the infiltrated tobacco was kept under constant light for 1.5–2 days. For chilling treatment, at 1.5–2 days post infiltration, the infiltrated tobacco leaves were detached from tobacco plants, kept in petri dishes with the moistened filter paper and incubated at 25 or 4 °C for overnight, respectively. Images were captured with a confocal laser scanning system (Leica, SP5 X, Leica Microsystems Inc., Buffalo Grove, IL, USA) and fluorescence microscope (Zeiss Axio-Plan, Carl Zeiss Microscopy, Thornwood, NY, USA). The fluorescence signals were detected at 510 nm (excitation at 488 nm) for GFP and at 610 nm (excitation at 587 nm) for mCherry.

## Results

### *AtGRXS17*-expressing tomato plants have enhanced chilling tolerance

**Figure 1:**
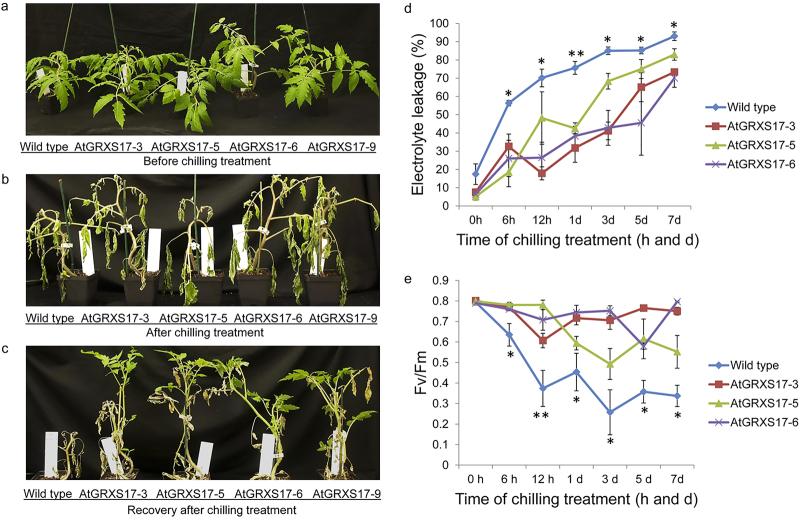
Effect of ectopically expressed *AtGRXS17* in tomato on chilling stress. (**a**) Phenotype of 4-week-old *AtGRXS17*-expressing and wild-type tomato plants before chilling treatment. (**b**) Phenotype of *AtGRXS17*-expressing and wild-type plants treated under 4 °C (day/night) for 3 weeks. (**c**) Two-weeks recovery after 3-week-chilling treatment. Electrolyte leakage (**d**) and chlorophyll fluorescence (**e**) of *AtGRXS17*-expressing and wild-type plants during chilling treatment. Four-week-old *AtGRXS17*-expressing and wild-type tomato plants grown at 24 °C/20 °C (day/night) were transferred to 4 °C. The leaves (the bottom second leaves) were sampled after 0 h, 6 h, 12 h, 1 days, 3 days, 5 days, and 7 days chilling treatment, respectively, and electrolyte leakage and chlorophyll fluorescence were analyzed. Data represent means ± SD from three independent biological replicates and were analyzed using Student’s *t-*test. Asterisks (*,**) represent statistically significant differences between wild-type and *AtGRXS17*-expressing lines (*P < 0.05, **P < 0.01).

**Figure 2:**
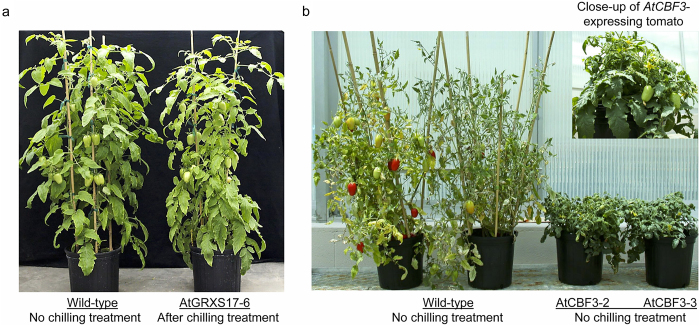
The phenotype of wild-type and *AtCBF3*-expressing tomato plants grown under normal growth conditions and *AtGRXS17*-expressing tomato plants recovered after being treated by chilling stress. (**a**) The *AtGRXS17*-expressing tomato plants showed normal growth and were indistinguishable from that of wild-type plants. The *AtGRXS17*-expressing tomato plants also do not appear to have adverse effects on fruit shape and size. (**b**) The phenotype of wild-type and *AtCBF3*-expressing tomato plants under normal growth conditions (24 °C/20 °C (day/night)).

Four tomato lines (*AtGRXS17*-3, -5, -6, and -9) that contain single *AtGRXS17* transgene insertions were selected from more than 20 independent transgenic lines generated in our previous studies,^28^ and the expression of *AtGRXS17* of the lines was verified by real-time qRT-PCR analysis (Supplementary Figure S1). Thirty plants each of homozygous *AtGRXS17*-expressing T2 generation plants from the four lines were subjected to chilling stress treatment. The growth and development of *AtGRXS17*-expressing tomato plants were visually indistinguishable from those of wild-type plants before chilling treatment (Figure 1A). While wild-type and *AtGRXS17*-expressing tomato plants both wilted after 3 weeks of chilling treatment (Figure 1B), *AtGRXS17*-expressing tomato plants appeared more vigorous in the 2-weeks recovery period under normal growth conditions (24 °C/20 °C day/night cycle) compared with wild-type plants (Figure 1C). The enhanced chilling stress tolerance of the three transgenic lines (*AtGRXS17*-3, -5, and -6, which show relatively higher expression level of *AtGRXS17* than that of *AtGRXS17*-9, Supplementary Figure S1) was further measured on the basis of electrolyte leakage and chlorophyll fluorescence. *AtGRXS17*-expressing plant leaves had lower electrolyte leakage, indicative of reduced disruption of cell membranes, when compared with wild-type plants after the chilling stress treatment (Figure 1D). Chlorophyll fluorescence of *AtGRXS17*-expressing plants, as measured by the Fv/Fm ratio (the maximum quantum efficiency of Photosystem II), was higher than that of wild-type plants (Figure 1E). After challenged by chilling stress, the growth and yield of the *AtGRXS17*-expressing tomatoes, which completely recovered, were observed to be indistinguishable from the wild-type plants under normal growth conditions^28^ (Figure 2A).

AtCBF3 is a transcription factor that confers cold tolerance.^9^ We generated the stress inducible *RD29A*::*AtCBF3*-expressing tomatoes as positive control lines (Supplementary Figure S2A; lines, *AtCBF3*-2 and -3) and compared basal (non-acclimated) chilling tolerance of the plants to *AtGRXS17*-expressing and wild-type lines. Thirty plants each of *AtCBF3-*expressing T2 generation plants from the two independent homozygous lines (*AtCBF3*-2 and -3) were subjected to chilling stress treatment. The leaves of wild-type plants were more severely wilted than those of *AtCBF3*- or *AtGRXS17*-expressing tomato plants (Supplementary Figure S2B), and the wild-type tomato plants showed the most visible damage in comparison with *AtCBF3*- or *AtGRXS17*-expressing tomato plants (Supplementary Figure S2C). The enhanced chilling stress tolerance was similar in both *AtCBF3*- and *AtGRXS17*-expressing lines after chilling stress. *AtCBF3*-expressing tomato plants, on the other hand, displayed dwarfing and a reduced number of fruits as compared to wild-type plants (Figure 2B).

### Ectopic expression of *AtGRXS17* reduces H_2_O_2_ accumulation and the effects of oxidative stress in tomato

**Figure 3:**
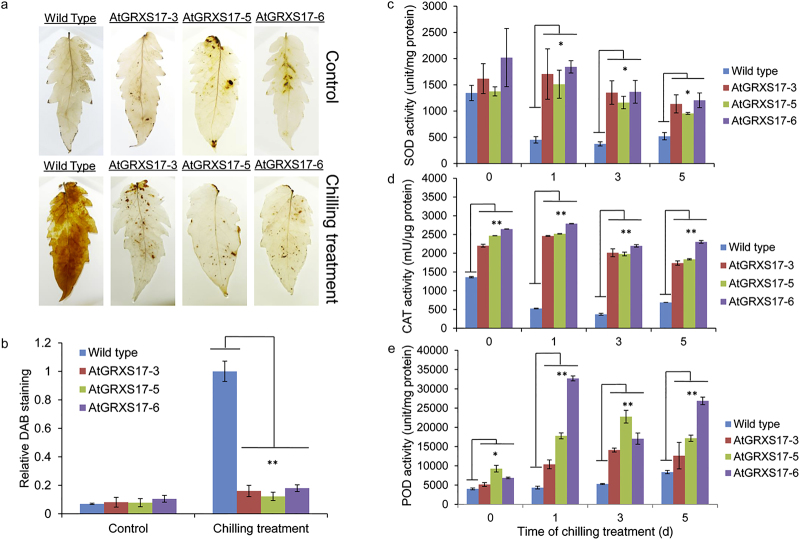
Effects of AtGRXS17 on H_2_O_2_ accumulation and the activities of ROS scavenging enzymes. (**a**) DAB staining in the terminal leaflet of the first fully expanded leaf of wild-type and *AtGRXS17*-expressing 4-week-old plants with or without chilling stress treatment. (**b**) Quantitative analysis of DAB staining. The relative intensity of DAB staining leaves was analyzed after being transformed to 256 grey scale images. Data are expressed as relative values based on wild-type plants treated at 4 °C as reference sample set as 1.0. Error bars represent the means ± SD (*n* = 3). The effects of *AtGRXS17* expression on superoxide dismutase (SOD) (**c**), catalase (CAT) (**d**), and guaiacol peroxidase (POD) (**e**) activity under normal and chilling stress conditions. Data represent means ± SD from three independent biological replicates and were analyzed using Student’s *t-*test. Asterisks (*,**) represent statistically significant differences between wild-type and *AtGRXS17*-expressing lines (*P < 0.05, **P < 0.01).

Chilling stress induces the production of ROS compounds including hydrogen peroxide (H_2_O_2_), which can cause oxidative stress damage to many organelles, membranes, proteins, DNA, and lipids.^45^ H_2_O_2_ accumulation was assayed using DAB staining of leaves from wild-type tomato plants and *AtGRXS17*-expressing tomato lines to examine how the expression of *AtGRXS17* in chilling-stressed tomatoes influences H_2_O_2_ accumulation. In the absence of chilling stress, leaves from wild-type and *AtGRXS17*-expressing tomato plants showed minimal DAB staining, indicating low H_2_O_2_ accumulation (Figure 3A, upper row). After 2–3 days of chilling treatment in the dark, substantial brown-staining material was detected in leaves of wild-type plants. In contrast, leaves of *AtGRXS17*-expressing plants showed less brown staining than those of the wild-type plants (Figure 3A, lower row), indicating less H_2_O_2_ accumulation in *AtGRXS17*-expressing tomato plants. Quantitative analysis of DAB staining density on the leaf surface showed that H_2_O_2_ accumulation was substantially lower in *AtGRXS17*-expressing leaves compared with that of the wild-type plants after the chilling treatment (Figure 3B). In addition, to determine if the selected transgenic lines have improved tolerance to oxidative stress, *AtGRXS17*-expressing and wild-type tomato seedlings were grown in MS media with or without MV, a pro-oxidant herbicide that stimulates formation of destructive ROS.^46^ *AtGRXS17*-expressing tomato seedlings displayed more vigorous growth visually and had longer primary root growth as compared to wild-type seedlings at 14 days of growth, indicating that AtGRXS17 ameliorates defective root growth and development due to oxidative stress (Supplementary Figure S3).

### Ectopic expression of *AtGRXS17* affects the activities of ROS scavenging enzymes in tomato

The possible roles of antioxidant enzymes in the transgenic lines were examined by measuring the activities of SOD, CAT, and POD. The activities of SOD, CAT, and POD were higher in all *AtGRXS17*-expressing lines than those of wild-type tomato plants in the first 5 days of chilling stress treatment (Figure 3C–3E). The activity of SOD in *AtGRXS17*-expressing lines and wild-type plants displayed no differences before chilling treatment (Figure 3C and 3D). After chilling treatment, all transgenic lines maintained higher SOD activity steadily in comparison to the wild-type plants that showed dramatically decreased SOD activity over 5 days of chilling treatment (Figure 3C). All transgenic lines showed greater CAT activity than that of the wild-type plants and maintained higher CAT activity over the 5 days under both normal and chilling stress conditions (Figure 3D). All transgenic lines also showed increased POD activity in comparison to the wild-type plants over 5 days under both normal and chilling stress conditions (Figure 3E).

**Figure 4:**
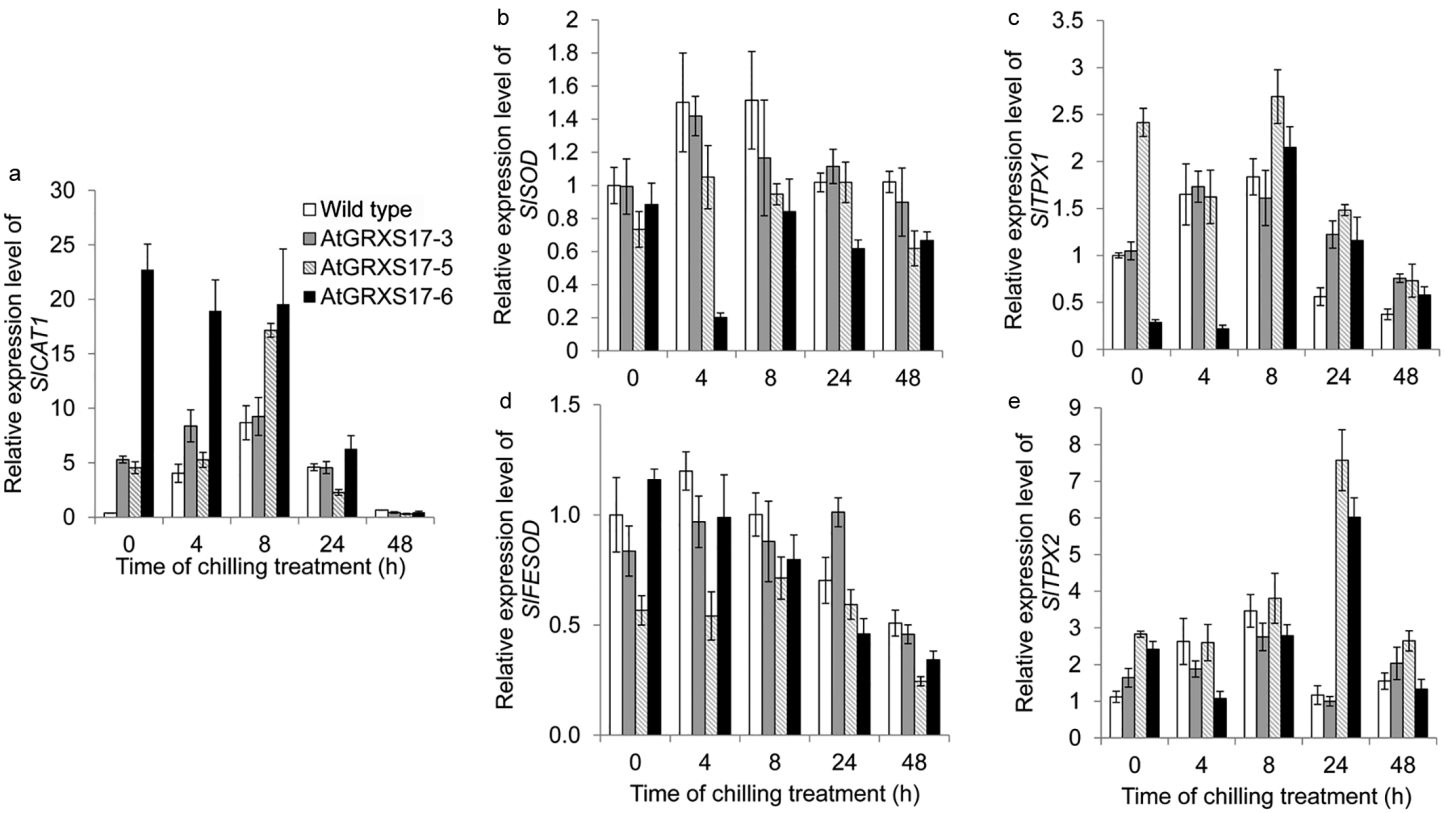
Effects of *AtGRXS17* expression on temporal expression patterns and levels of genes encoding ROS scavenging enzymes under chilling stress. Relative mRNA levels of *SlCAT1* (**a**), *SlSOD* (**b**), *SlTPX1* (**c**), *SlFESOD* (**d**), and *SlTPX2* (**e**) in 4-week-old wild-type and *AtGRXS17*-expressing tomato plants were analyzed after being treated at 4 °C for 0, 4, 8, 24, and 48 h, respectively. Data represent means ± SD from three independent biological replicates.

Antioxidant enzyme activities and transcript levels of their respective genes may not correlate tightly.^47^ The transcription levels of antioxidant enzyme genes *SlCAT1*, *SlSOD*, *SlFESOD*, *SlTPX1* and *SlTPX2* (Supplementary Table S1), and enzyme activity levels were measured during chilling stress (Figure 4). The *SlCAT1* transcript levels in all *AtGRXS17*-expressing lines were higher than those of wild-type tomato plants before chilling treatment (Figure 4A). During chilling stress, the *SlCAT1* transcript levels in both wild-type and *AtGRXS17*-expressing lines were increased in first 8 h treatment and then returned to resting levels at 48 h (Figure 4A). The overall expression patterns and levels of *SlSOD, SlFESOD*, *SlTPX1,* and *SlTPX2* were very similar in both wild-type and *AtGRXS17*-expressing lines, showing slightly increased transcripts in first 8 h and then decreasing within 48 h under chilling stress, though variation of gene transcript levels among *AtGRXS17*-expressing lines was observed (Figure 4B–4E). These results indicate that *AtGRXS17* expression in chilling-stressed tomatoes affects activity and stability, but not the transcript level of the antioxidant enzymes.

### Effect of ectopic expression of *AtGRXS17* on the accumulation of proline and soluble sugars under chilling stress

**Figure 5:**
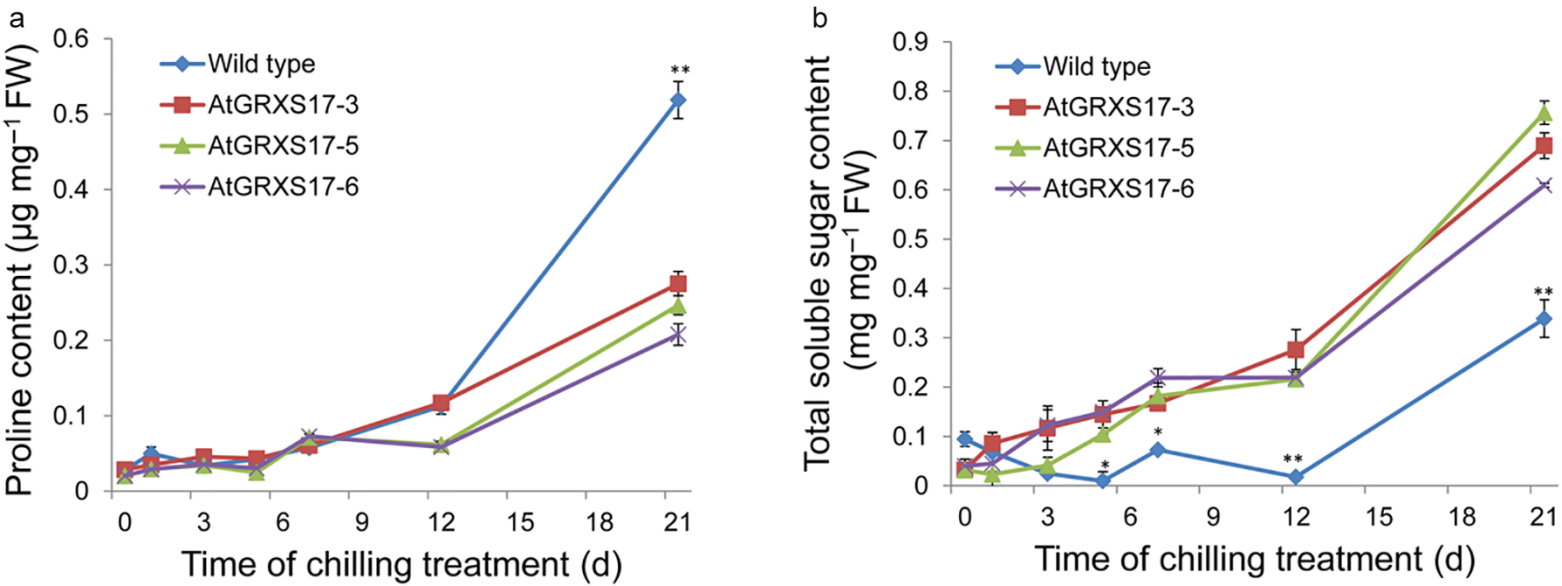
Effects of ectopic expressing *AtGRXS17* in tomato on proline and soluble sugar accumulation under chilling stress. (**a**) The proline accumulation of wild-type and *AtGRXS17*-expressing tomato plants after being treated at 4 °C for 0, 1, 3, 5, 7, 12, and 21 days, respectively. (**b**) The total soluble sugar accumulation of wild-type and *AtGRXS17*-expressing tomato plants after being treated at 4 °C for 0, 1, 3, 5, 7, 12, and 21 days, respectively. Error bars represent the means ± SD (*n* = 3) and were analyzed using Student’s *t-*test. Asterisks (*,**) represent statistically significant differences between wild-type and *AtGRXS17*-expressing lines (*P < 0.05, **P < 0.01).

An increase in proline content was observed in both wild-type and *AtGRXS17*-expressing tomato plants upon exposure to chilling stress. Proline content was not different between wild-type and *AtGRXS17*-expressing tomato plants during the first 12 days of chilling treatment. However, after 21 days of chilling treatment, the proline content of wild-type plants was higher than that of transgenic plants (Figure 5A), indicating that the enhanced chilling tolerance of *AtGRXS17*-expressing tomato plants was not due to proline accumulation. On the other hand, soluble sugar content in tomato leaves from wild-type and *AtGRXS17*-expressing plants had no difference in total soluble sugar content before chilling treatment and higher sugar content in all *AtGRXS17*-expressing lines as compared with wild-type tomato plants, exhibiting 2- to 3-fold elevated content after 5 days of chilling treatment (Figure 5B).

### AtGRXS17 accumulates in the nucleus during chilling stress

**Figure 6:**
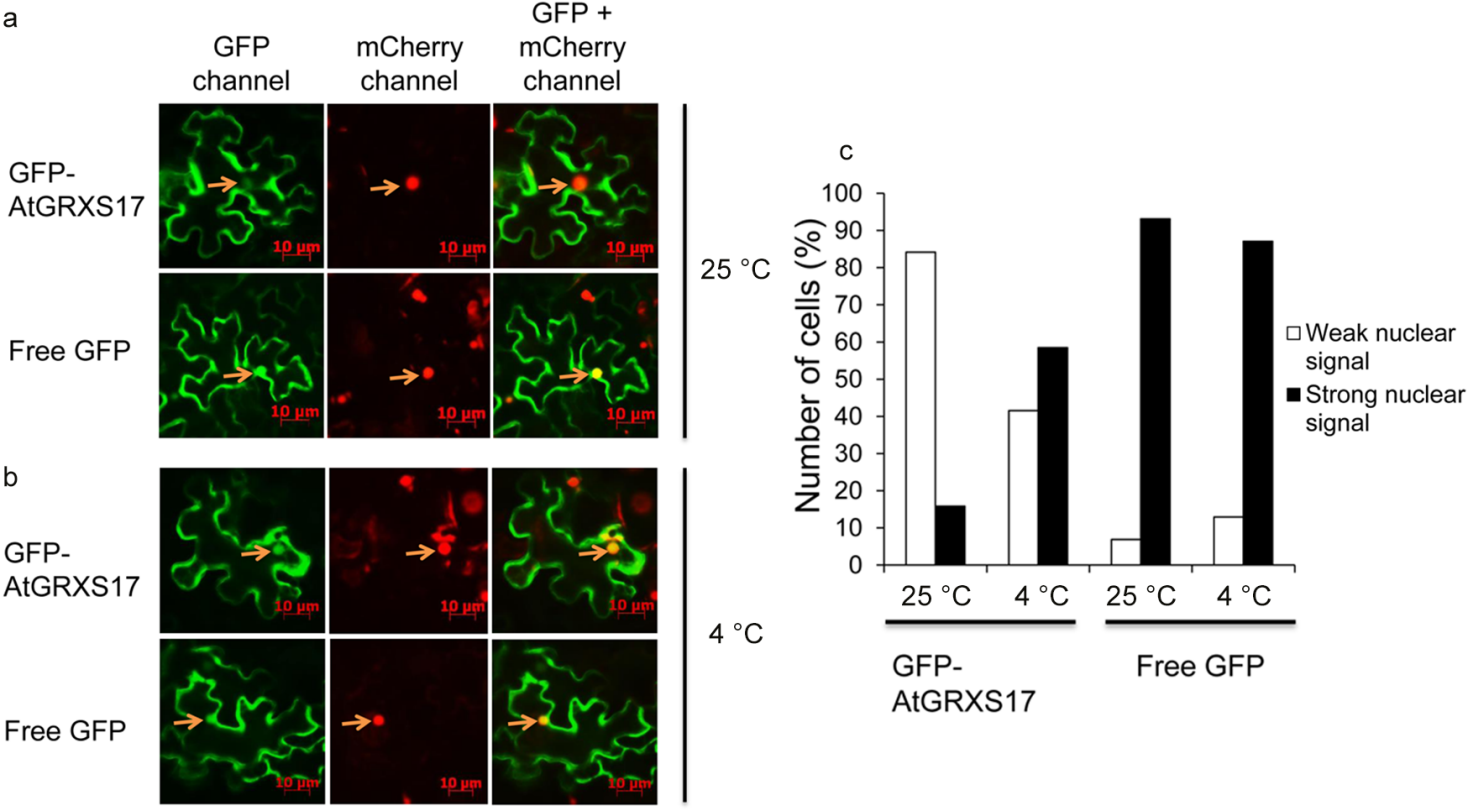
Subcellular localization of AtGRXS17. (**a**) Transient expression of *GFP-AtGRXS17* and free *GFP* in tobacco epidermal cells were imaged after being treated at 25 °C overnight, respectively. Scale bars = 10 µm. The arrows highlight the nuclei. (**b**) Transient expression of *GFP-AtGRXS17* and free *GFP* in tobacco epidermal cells after being treated at 4 °C overnight. (**c**) The numbers of cells (%) with weak nuclear signals or strong nuclear signals. AtGRXS17::GFP: 16 cells with GFP signal and 85 cells without GFP signal in the nucleus at 25 °C. Free GFP: 95 cells with GFP signal and 7 cells without GFP signal in the nucleus at 25 °C. AtGRXS17::GFP: 121 cells with GFP signal and 86 cells without GFP signal in the nucleus at 4 °C. Free GFP: 27 cells with GFP signal and 4 cells without GFP signal in the nucleus at 4 °C.

The subcellular localization of AtGRXS17 protein in plant cells with or without chilling stress treatment was examined using a version of the *AtGRXS17* gene that was fused to the C-terminus of *GFP* gene and transiently expressed under the control of the *CaMV 35S* promoter (*35S::GFP-AtGRXS17*) in tobacco leaf epidermal cells. A vector harboring *35S::mCherry::NLS* (the mCherry red fluorescent protein linked to a nuclear localization signal) was used as a control for nuclear localization in transient co-expression assays (Figure 6A and 6B, middle). *GFP* gene was also expressed under the control of the *35S* promoter as a control for free GFP localization (Figure 6A and 6B, lower panel). Under normal growth conditions (25 °C), the GFP-AtGRXS17 fusion protein was primarily detected in the cytoplasm and the nuclei (Figure 6A left, upper panel). Eighty-five percent (85 out of 101) of the cells had fluorescence signals detected in the cytoplasm with weaker signals in the nucleus, while 15.8% (16 out of 101) of cells having stronger signals detected in the nucleus (Figure 6C). In response to chilling stress (overnight at 4 °C), the GFP-AtGRXS17 fusion protein emitted strong fluorescence signals in the nuclei (Figure 6B left, upper panel). Forty-two percent (86 out of 207) cells having fluorescence signals detected in the cytoplasm with weaker signals in the nuclei, while 58.4% (121 out of 207) cells had stronger signals detected in the nuclei (Figure 6C), indicating that AtGRXS17 accumulates in the nucleus during chilling stress. In contrast, free GFP was localized in nuclei independent of chilling treatment (Figure 6A and 6B, lower panel and Figure 6C).

### Ectopic expression of *AtGRXS17* in tomato does not alter expression patterns of *SlCBF1* and *CBF* target genes under chilling stress

**Figure 7:**
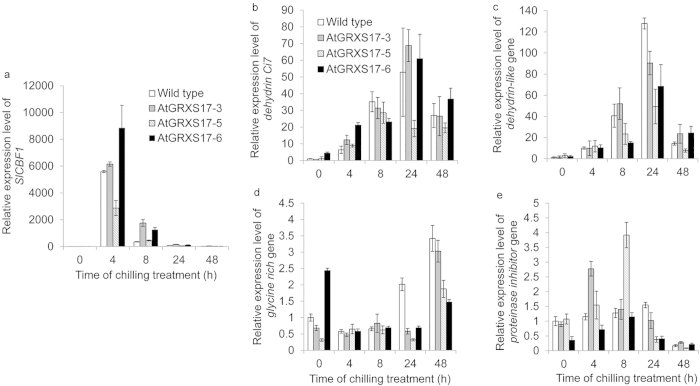
Expression patterns and levels of *SlCBF1* and four *CBF* target genes affected by *AtGRXS17* expression under chilling stress. Relative mRNA levels of *SlCBF1* (**a**), *dehyrin Ci7* gene (**b**), *dehydrin-like* gene (**c**), *glycine rich* gene (**d**), and *proteinase inhibitor* gene (**e**) in 4-week-old wild-type and *AtGRXS17*-expressing tomato plants were analyzed after being treated at 4 °C for 0, 4, 8, 24, and 48 h, respectively. Data represent means ± SD from three independent biological replicate.

The effect of *AtGRXS17* ectopic expression on the response of *SlCBF1* and four *CBF* target genes (*dehydrin Ci7*, *dehydrin-like*, *proteinase inhibitor,* and *glycine-rich*; Supplementary Table S1) to chilling stress in tomato plants was measured. *SlCBF1* expression levels in both wild-type and *AtGRXS17*-expressing lines peaked at 4 h after chilling stress and returned rapidly to resting levels (Figure 7A). No differences in overall expression patterns and levels were observed between wild-type and *AtGRXS17*-expressing lines, although variation of gene expression levels among *AtGRXS17*-expressing lines was observed as either higher or lower *SlCBF1* expression level compared to wild-type plants (Figure 7A). *Dehydrin Ci7* and *Dehydrin-like* expression levels in both wild-type and *AtGRXS17*-expressing lines were elevated and peaked at 24 h after chilling stress and then decreased at 48 h (Figure 7B and 7C). *Glycine-rich* expression remained at a constant level and then increased at 48 h in both wild-type and *AtGRXS17*-expressing lines, while *proteinase inhibitor* expression levels in both wild-type and *AtGRXS17*-expressing lines were increased slowly for the first 8 h and then decreased at 24 h (Figure 7D and 7E, respectively). Real-time qRT-PCR analysis showed that the overall expression patterns and levels of *SlCBF1* and four *CBF* target genes are similar between wild-type and *AtGRXS17*-expressing lines after chilling stress, indicating that the chilling tolerance in *AtGRXS17*-expressing tomato plants does not depend on the CBF pathway.

## Discussion

AtGRXS17 is a member of a family of global and conserved heat stress-responsive factor and confers thermotolerance in both yeast and plant species.^28^ In tomato, while endogenous *SlGRXS17* was expressed in all tissues, *SlGRXS17* expression was not induced by chilling stress for different time periods (Supplementary Figure S4A and 4B). However, ectopically expressed *AtGRXS17* in tomato modulated a number of cold-responsive components to suppress chilling-induced oxidative damage and enhance chilling tolerance. At the same time, AtGRXS17-dependent chilling tolerance had features that indicate the tolerance is independent of the CBF pathway.

ROSs are known to accumulate during various abiotic stresses, causing damage to macromolecules and ultimately to cellular structure.^13,48–51^ On the other hand, ROSs are key regulators of growth, development, and defense pathways.^52^ Thus, the ROS and redox state must be tightly regulated by ROS-scavenging and ROS-producing systems. Many studies demonstrated that GRXs play roles in regulating redox homeostasis.^53–56^ GRXs are oxidoreductase enzymes that are capable of mediating reversible reduction of their substrate proteins in the presence of GSH (ref. 57), therefore maintaining and regulating the cellular redox state and redox-dependent signaling pathways.^19^ Our working hypothesis in this study is that ectopic expression of *AtGRXS17* in tomato plants may play an important role in the coordination of signaling and scavenging of ROS through improving the adjustment capability of redox status of plant cells and balancing the damaging and signaling of ROS, resulting in enhanced tolerance to chilling stress. Indeed, *AtGRXS17*-expressing tomato plants showed higher chilling tolerance as compared to the wild-type tomato plants, partially due to preventing photo-oxidation of chlorophyll and reducing the oxidative damage under chilling stress. Furthermore, our MV treatment data provide direct evidence that AtGRXS17 relieves the defective growth of primary roots correlated with increased accumulation of ROS. *AtGRXS17*-expressing tomato plants also displayed higher antioxidant enzyme activities, less H_2_O_2_ accumulation in the *in vitro* test of tomato leaves under chilling stress, more vigorous growth and significantly longer root length as compared to the wild-type plants under oxidative stress, indicating that AtGRXS17 is an important component of the cellular ROS-scavenging/antioxidant system.

To adapt to various environmental stresses, plants have evolved specific enzymatic antioxidants such as CAT, SOD, and POD to protect themselves from oxidative stress. Interestingly, *AtGRXS17*-expressing tomato plants maintained significantly higher activities of CAT, SOD, and POD steadily as compared with wild-type tomato plants over 5 days under chilling stress, while transcript levels of those antioxidant genes in both wild-type and *AtGRXS17*-expressing lines were nearly identical. The results revealed that AtGRXS17-mediated chilling tolerance was associated with increased activities and stabilities, rather than transcript levels, of the antioxidant enzymes. It is possible that AtGRXS17 is able to protect CAT, SOD, and POD as target proteins by thiol–disulfide exchange. Previous studies indicated that there are several proteins identified as GRX targets, especially in response to oxidative stress conditions. The bovine Cu, Zn SOD, as nonplant GRX targets, have been found to be glutathiolated and are thus potential targets of GRX for deglutathiolation.^58^ It was also reported that CAT is one of GRX-interacting proteins targeted by Poplar GRX C1 (ref. 20). Recently, numerous proteins have been identified as GRX substrate proteins by using Reversibly Oxidized Cysteine Detector (ref. 59). CAT isozyme 2 and SOD belong to these targeted proteins.^59^ Another implication is that AtGRXS17 may protect CAT, SOD, and POD indirectly by increasing the production of total soluble sugars under chilling stress. It has been reported that soluble sugars interact with proteins and membranes through hydrogen bonding, thereby preventing protein denaturation.^60^ In fact, we found that *AtGRXS17*-expressing tomato plants had a significantly higher amount of soluble sugars as compared with wild-type plants, which may contribute to the protection of CAT, SOD, and POD under chilling stress.

Proline is a known protectant in abiotic stress and oxidative damage.^61–63^ Interestingly, our results suggest that the proline content of transgenic lines was lower than that of wild-type plants after 21-days chilling treatment. Despite this, both follow a similar trend as proline content was increased in both wild-type and *AtGRXS17*-expressing tomato plants over the treatment period. It is possible that proline accumulation is induced by ROS signaling. In *Arabidopsis*, the expression of *AtP5CS2* was up-regulated by ROS that correspondingly resulted in accumulation of proline.^64^ It is also reported that proline accumulation is induced by H_2_O_2_ in rice seedlings.^65^ Therefore, the lower proline content may be due to less ROS accumulation in the transgenic plants. Unlike proline, soluble sugar content was higher in the *AtGRXS17*-expressing plants during chilling stress. Soluble sugars act as osmoprotectants to prevent cellular membrane damage caused by dehydration.^66,67^ It is also reported that soluble sugars contribute to ROS scavenging by supporting NADPH-producing metabolic pathways.^68^ For instance, glucose has been shown to protect certain mammalian cell types from cytotoxicity of H_2_O_2_ (ref. 69). Besides, glucose is involved in synthesis of antioxidant compounds as well as acts as a carbonic precursor for some amino acids, which are involved in synthesis of glutathione.^63,70^ Proteomic analyses have identified a large number of TRX and GRX target candidates that are involved in sugar and starch synthesis.^71^ Thus, AtGRXS17-mediated regulation of the sugar content may partially contribute to the chilling stress tolerance.

The subcellular localization changes of AtGRXS17 are consistent with the changes observed during heat stress.^28^ This translocation could be caused by the accumulation of ROS under stress conditions. Protein nuclear translocation in response to ROS has been intensively studied in mammalian cells.^72^ The nuclear pool of DJ-1, the protein that protects neurons from oxidative stress, dramatically increases after treatment with H_2_O_2_, whereas the nuclear translocation of DJ-1 can be blocked by applying antioxidants.^72^ In addition, glyceraldehyde-3-phosphate dehydrogenase, a protein which plays roles in apoptosis and oxidative stress, is translocated to the nucleus in response to H_2_O_2_ treatment.^73^ Interestingly, AtGRXS17 does not have predicted nuclear targeting signals; therefore, AtGRXS17 nuclear translocation may be facilitated by other proteins under chilling stress condition.

One possible importance of translocation of AtGRXS17 into the nucleus is that AtGRXS17 may interact with some transcription factors. Previous studies have demonstrated that GRXs can interact with transcription factors in plants. For example, the *Arabidopsis* ROXY1, a CC-type GRX, has been reported to control petal development by interacting with TGA transcription factors.^74^ Similarly, the maize ROXY1 ortholog MSCA1 also interacts with FEA4, a maize TGA transcription factor, and the interaction may contribute to the maize shoot apical meristem regulation.^75,76^ Furthermore, the yeast ortholog protein of AtGRXS17, GRX3, has been reported to interact with Aft1, a transcription factor that regulates iron homeostasis.^77^ Recently, AtGRXS17 has been shown to interact with a nuclear transcription factor, NF-YC11/NC2α to maintain its redox state, and this interaction may contribute to the shoot apical meristem maintenance.^27^ All of these studies indicate that the GRXs could play important roles in the nucleus through interaction with transcription factors. Another possible importance of translocation of AtGRXS17 into the nucleus is that the protein may function to protect DNA from ROS toxicity under abiotic stress conditions. ROSs are a major source of DNA damage,^49^ which leads to malfunctions or complete inactivation of encoded proteins.^78^ Therefore, AtGRXS17 may translocate to nuclei to interact with transcription factors that activate expression of stress-related genes or further protect DNA from ROS damage under chilling stress conditions.

The CBF/DREB1 pathway is a well-characterized cold response pathway,^79^ and various approaches have been proposed to improve cold (freezing and/or chilling) tolerance by manipulating *CBF* expression across different species.^80–82^ Although the induction of *CBFs* is one of the predominant responses to cold stress, over-accumulation of CBFs disrupts the regular biological processes of plants.^83^ Indeed, constitutive expression of either *AtCBF3* or *SlCBF1* in both *Arabidopsis* and tomato plants results in stunted growth and a significant yield penalty.^7,9^ Even though the stress inducible *RD29A* promoter was used to minimize the negative effects on plant growth in this study, the *AtCBF3*-expressing tomato plants still displayed stunted growth and a reduced yield under normal growth conditions (24/20 °C (day/night)) (Figure 2B). The lack of stunted growth in plants expressing *AtGRXS17* suggests that manipulation of AtGRXS17 may be a useful alternative to CBFs to improve chilling tolerance across different species.

Unlike *Arabidopsis*, which includes cold-induced expression of three homologous *CBFs* (*CBF1*, *2* and *3*) and a large and diverse *CBF* regulon, the *CBF1* ortholog (*SlCBF1*) in tomato is the only cold-inducible *CBF*. In addition, transcriptome analysis using a cDNA microarray covering approximately 25% of the tomato genome found only four candidate genes for a CBF regulon induced by chilling stress and over-expression of *SlCBF1* or *AtCBF3* in tomato plants.^7^ Our results demonstrate that ectopic expression of *AtGRXS17* in tomato does not alter expression patterns and levels of *SlCBF1* and those four *CBF* target genes compared to wild-type plants under chilling stress, indicating AtGRXS17-mediated chilling tolerance may not be associated with part of the CBF-dependent transcriptional pathway. Several studies have also reported that chilling tolerance in plants is attributed to cold-responsive pathways other than the CBF cold-responsive pathway. For instance, HOS9, an *Arabidopsis* homeodomain transcription factor, is involved in maintaining freezing tolerance through a constitutive pathway instead of CBF cold-responsive pathway.^84^ The mutations in ESKIMO1 confers enhanced freezing tolerance by regulating salt and osmotic stress or ABA responsive genes rather than those of the CBF regulon.

Taken together, our results suggest that ectopic expression of *AtGRXS17* enhances the chilling tolerance of tomato plants. Minimized photo-oxidation of chlorophyll, reduced oxidative damage of cell membranes, increased activities of antioxidant enzymes such as CAT, SOD, and POD, accumulation of osmoprotectant soluble sugars, and reduced accumulation of H_2_O_2_ are all associated with the enhanced chilling tolerance. Due to the conserved function of GRXs in plant species, manipulation of GRXs across different species may prove to be an invaluable approach for plant breeders to improve chilling stress tolerance without any adverse effects on plant growth and development.

## References

[bib1] Cruz RP, Sperotto RA, Cargnelutti D, Adamski JM, FreitasTerra T, Fett JP. Avoiding damage and achieving cold tolerance in rice plants. Food Energy Secur 2013; 2: 96–119.

[bib2] Weiss J, Egea-Cortines M. Transcriptomic analysis of cold response in tomato fruits identifies dehydrin as a marker of cold stress. J Appl Genet 2009; 50: 311–319. 19875881 10.1007/BF03195689

[bib3] Chinnusamy V, Schumaker K, Zhu JK. Molecular genetic perspectives on cross-talk and specificity in abiotic stress signalling in plants. J Exp Bot 2004; 55: 225–236. 14673035 10.1093/jxb/erh005

[bib4] Fowler S, Thomashow MF. Arabidopsis transcriptome profiling indicates that multiple regulatory pathways are activated during cold acclimation in addition to the CBF cold response pathway. Plant Cell 2002; 14: 1675–1690. 12172015 10.1105/tpc.003483 PMC151458

[bib5] Thomashow MF. Molecular basis of plant cold acclimation: insights gained from studying the CBF cold response pathway. Plant Physiol 2010; 154: 571–577. 20921187 10.1104/pp.110.161794 PMC2948992

[bib6] Carvallo MA, Pino MT, Jeknić Z et al. A comparison of the low temperature transcriptomes and CBF regulons of three plant species that differ in freezing tolerance: *Solanum commersonii*, *Solanum tuberosum*, and *Arabidopsis thaliana*. J Exp Bot 2011; 62: 3807–3819. 21511909 10.1093/jxb/err066 PMC3134341

[bib7] Zhang X, Fowler SG, Cheng H et al. Freezing-sensitive tomato has a functional CBF cold response pathway, but a CBF regulon that differs from that of freezing-tolerant *Arabidopsis*. Plant J 2004; 39: 905–919. 15341633 10.1111/j.1365-313X.2004.02176.x

[bib8] Hsieh TH, Lee JT, Charng YY, Chan MT. Tomato plants ectopically expressing *Arabidopsis* CBF1 show enhanced resistance to water deficit stress. Plant Physiol 2002; 130: 618–626. 12376629 10.1104/pp.006783 PMC166591

[bib9] Kasuga M, Liu Q, Miura S, Yamaguchi-Shinozaki K, Shinozaki K. Improving plant drought, salt, and freezing tolerance by gene transfer of a single stress-inducible transcription factor. Nat Biotechnol 1999; 17: 287–291. 10096298 10.1038/7036

[bib10] Pino MT, Skinner JS, Park EJ et al. Use of a stress inducible promoter to drive ectopic AtCBF expression improves potato freezing tolerance while minimizing negative effects on tuber yield. Plant Biotech J 2007; 5: 591–604. 10.1111/j.1467-7652.2007.00269.x 17559519

[bib11] Jaglo KR, Kleff S, Amundsen KL et al. Components of the *Arabidopsis* C-repeat/dehydration-responsive element binding factor cold-response pathway are conserved in *Brassica napus* and other plant species. Plant Physiol 2001; 127: 910–917. 11706173 PMC129262

[bib12] Achard P, Gong F, Cheminant S, Alioua M, Hedden P, Genschik P. The cold-inducible CBF1 factor-dependent signaling pathway modulates the accumulation of the growth-repressing DELLA proteins via its effect on gibberellin metabolism. Plant Cell 2008; 20: 2117–2129. 18757556 10.1105/tpc.108.058941 PMC2553604

[bib13] Jaspers P, Kangasjärvi J. Reactive oxygen species in abiotic stress signaling. Physiol Plant 2010; 138: 405–413. 20028478 10.1111/j.1399-3054.2009.01321.x

[bib14] Gill SS, Tuteja N. Reactive oxygen species and antioxidant machinery in abiotic stress tolerance in crop plants. Plant Physiol Biochem 2010; 48: 909–930. 20870416 10.1016/j.plaphy.2010.08.016

[bib15] Suzuki N, Mittler R. Reactive oxygen species and temperature stresses: a delicate balance between signaling and destruction. Physiol Plant 2006; 126: 45–51.

[bib16] Foyer CH, Noctor G. Redox homeostasis and antioxidant signaling: a metabolic interface between stress perception and physiological responses. Plant Cell 2005; 17: 1866–1875. 15987996 10.1105/tpc.105.033589 PMC1167537

[bib17] Rouhier N, Lemaire SD, Jacquot JP. The role of glutathione in photosynthetic organisms: emerging functions for glutaredoxins and glutathionylation. Annu Rev Plant Biol 2008; 59: 143–166. 18444899 10.1146/annurev.arplant.59.032607.092811

[bib18] Cheng NH, Liu JZ, Liu X et al. *Arabidopsis* monothiol glutaredoxin, AtGRXS17, is critical for temperature-dependent postembryonic growth and development via modulating auxin response. J Biol Chem 2011; 286: 20398–20406. 21515673 10.1074/jbc.M110.201707 PMC3121514

[bib19] Lillig CH, Berndt C, Holmgren A. Glutaredoxin systems. Biochim Biophys Acta 2008; 1780: 1304–1317. 18621099 10.1016/j.bbagen.2008.06.003

[bib20] Rouhier N, Villarejo A, Srivastava M et al. Identification of plant glutaredoxin targets. Antioxid Redox Signal 2005; 7: 919–929. 15998247 10.1089/ars.2005.7.919

[bib21] Shelton MD, Chock PB, Mieyal JJ. Glutaredoxin: role in reversible protein s-glutathionylation and regulation of redox signal transduction and protein translocation. Antioxid Redox Signal 2005; 7: 348–366. 15706083 10.1089/ars.2005.7.348

[bib22] Sundaram S, Rathinasabapathi B. Transgenic expression of fern *Pteris vittata* glutaredoxin PvGrx5 in *Arabidopsis thaliana* increases plant tolerance to high temperature stress and reduces oxidative damage to proteins. Planta 2010; 231: 361–369. 19936779 10.1007/s00425-009-1055-7

[bib23] Sundaram S, Wu S, Ma LQ, Rathinasabapathi B. Expression of a *Pteris vittata* glutaredoxin PvGRX5 in transgenic *Arabidopsis thaliana* increases plant arsenic tolerance and decreases arsenic accumulation in the leaves. Plant Cell Environ 2009; 32: 851–858. 19236608 10.1111/j.1365-3040.2009.01963.x

[bib24] La Camera S, L’Haridon F, Astier J et al. The glutaredoxin ATGRXS13 is required to facilitate *Botrytis cinerea* infection of *Arabidopsis thaliana* plants. Plant J 2011; 68: 507–519. 21756272 10.1111/j.1365-313X.2011.04706.x

[bib25] Laporte D, Olate E, Salinas P, Salazar M, Jordana X, Holuigue L. Glutaredoxin GRXS13 plays a key role in protection against photooxidative stress in *Arabidopsis*. J Exp Bot 2011: 63: 503–515. 21963612 10.1093/jxb/err301 PMC3245481

[bib26] Cheng NH, Zhang W, Chen WQ et al. A mammalian monothiol glutaredoxin, Grx3, is critical for cell cycle progression during embryogenesis. FEBS J 2011; 278: 2525–2539. 21575136 10.1111/j.1742-4658.2011.08178.x PMC4038268

[bib27] Knuesting J, Riondet C, Maria C et al. *Arabidopsis* glutaredoxin S17 and its partner NF-YC11/NC2α contribute to maintenance of the shoot apical meristem under long-day photoperiod. Plant Physiol 2015; 167: 1643–1658. 25699589 10.1104/pp.15.00049 PMC4378178

[bib28] Wu Q, Lin J, Liu JZ et al. Ectopic expression of *Arabidopsis* glutaredoxin AtGRXS17 enhances thermotolerance in tomato. Plant Biotech J 2012; 10: 945–955. 10.1111/j.1467-7652.2012.00723.x 22762155

[bib29] Feng Y, Cao CM, Vikram M et al. A three-component gene expression system and its application for inducible flavonoid overproduction in transgenic *Arabidopsis thaliana*. PLoS One 2011; 6: e17603. 21408135 10.1371/journal.pone.0017603 PMC3050924

[bib30] Holsters M, De Waele D, Depicker A, Messens E, Van Montagu M, Schell J. Transfection and transformation of *Agrobacterium tumefaciens*. Mol Gen Genet 1978; 163: 181–187. 355847 10.1007/BF00267408

[bib31] Murashige T, Skoog F. A revised medium for rapid growth and bio assays with tobacco 30. tissue cultures. Physiol Plantarum 1962; 15: 473–497.

[bib32] Park SH, Morris JL, Park JE, Hirschi KD, Smith RH. Efficient and genotype-independent Agrobacterium-mediated tomato transformation. J Plant Physiol 2003; 160: 1253–1257. 14610894 10.1078/0176-1617-01103

[bib33] Lovdal T, Lillo C. Reference gene selection for quantitative real-time PCR normalization in tomato subjected to nitrogen, cold, and light stress. Anal Biochem 2009; 387: 238–242. 19454243 10.1016/j.ab.2009.01.024

[bib34] Oh MM, Trick HN, Rajashekar CB. Secondary metabolism and antioxidants are involved in environmental adaptation and stress tolerance in lettuce. J Plant Physiol 2009; 166: 180–191. 18562042 10.1016/j.jplph.2008.04.015

[bib35] Bindschedler LV, Dewdney J, Blee KA et al. Peroxidase-dependent apoplastic oxidative burst in *Arabidopsis* required for pathogen resistance. Plant J 2006; 47: 851–863. 16889645 10.1111/j.1365-313X.2006.02837.x PMC3233234

[bib36] Thordal-Christensen H, Zhang Z, Wei Y, Collinge DB. Subcellular localization of H_2_O_2_ in plants. H_2_O_2_ accumulation in papillae and hypersensitive response during the barley—powdery mildew interaction. Plant J 1997; 11: 1187–1194.

[bib37] Beyer WF Jr, Fridovich I. Assaying for superoxide dismutase activity: some large consequences of minor changes in conditions. Anal Biochem 1987; 161: 559–566. 3034103 10.1016/0003-2697(87)90489-1

[bib38] Maehly A, Chance B. Catalases and peroxidases. Methods Biochem Anal 1954; 1: 357–424. 13193536 10.1002/9780470110171.ch14

[bib39] Ábrahám E, Hourton-Cabassa C, Erdei L, Szabados L. Methods for determination of proline in plants. Methods Mol Biol 2010; 639: 317–331. 20387056 10.1007/978-1-60761-702-0_20

[bib40] Yemm EW, Willis AJ. The estimation of carbohydrates in plant extracts by anthrone. Biochem J 1954; 57: 508–514. 13181867 10.1042/bj0570508 PMC1269789

[bib41] Sparkes IA, Runions J, Kearns A, Hawes C. Rapid, transient expression of fluorescent fusion proteins in tobacco plants and generation of stably transformed plants. Nat Protoc 2006; 1: 2019–2025. 17487191 10.1038/nprot.2006.286

[bib42] Cheng NH, Liu JZ, Brock A, Nelson RS, Hirschi KD. AtGRXcp, an *Arabidopsis* chloroplastic glutaredoxin, is critical for protection against protein oxidative damage. J Biol Chem 2006; 281: 26280–26288. 16829529 10.1074/jbc.M601354200

[bib43] Karimi M, Inzé D, Depicker A. GATEWAY^™^ vectors for Agrobacterium-mediated plant transformation. Trends Plant Sci 2002; 7: 193–195. 11992820 10.1016/s1360-1385(02)02251-3

[bib44] Shigaki T, Kole M, Ward JM, Sze H, Hirschi KD. Cre-loxP recombination vectors for the expression of Riken *Arabidopsis* full-length cDNAs in plants. BioTechniques 2005; 39: 301–302, 304. 16206900 10.2144/05393BM01

[bib45] Apel K, Hirt H. Reactive oxygen species: metabolism, oxidative stress, and signal transduction. Annu Rev Plant Biol 2004; 55: 373–399. 15377225 10.1146/annurev.arplant.55.031903.141701

[bib46] Foyer CH, Noctor G. Redox regulation in photosynthetic organisms: signaling, acclimation, and practical implications. Antioxid Redox Signal 2009; 11: 861–905. 19239350 10.1089/ars.2008.2177

[bib47] Stitt M, Gibon Y. Why measure enzyme activities in the era of systems biology? Trends Plant Sci 2014; 19: 256–265. 24332227 10.1016/j.tplants.2013.11.003

[bib48] Fridovich I. Biological effects of the superoxide radical. Arch Biochem Biophys 1986; 247: 1–11. 3010872 10.1016/0003-9861(86)90526-6

[bib49] Imlay JA, Linn S. DNA damage and oxygen radical toxicity. Science 1988; 240: 1302–1309. 3287616 10.1126/science.3287616

[bib50] Kelvin JAD. Protein damage and degradation by oxygen radicals. J Biol Chem 1987; 262: 9895–9901. 3036875

[bib51] Møller IM, Jensen PE, Hansson A. Oxidative modifications to cellular components in plants. Annu Rev Plant Biol 2007; 58: 459–481. 17288534 10.1146/annurev.arplant.58.032806.103946

[bib52] Mittler R, Vanderauwera S, Gollery M, Van Breusegem F. Reactive oxygen gene network of plants. Trends Plant Sci 2004; 9: 490–498. 15465684 10.1016/j.tplants.2004.08.009

[bib53] Rouhier N, Gelhaye E, Jacquot JP. Glutaredoxin-dependent peroxiredoxin from poplar protein-protein interaction and catalytic mechanism. J Biol Chem 2002; 277: 13609–13614. 11832487 10.1074/jbc.M111489200

[bib54] Rouhier N, Vlamis-Gardikas A, Lillig CH et al. Characterization of the redox properties of poplar glutaredoxin. Antioxid Redox Signal 2003; 5: 15–22. 12626113 10.1089/152308603321223504

[bib55] Sha S, Minakuchi K, Higaki N et al. Purification and characterization of glutaredoxin (thioltransferase) from rice (*Oryza sativa* L.). J Biochem 1997; 121: 842–848. 9192723 10.1093/oxfordjournals.jbchem.a021663

[bib56] Tsukamoto S, Morita S, Hirano E, Yokoi H, Masumura T, Tanaka K. A novel cis-element that is responsive to oxidative stress regulates three antioxidant defense genes in rice. Plant Physiol 2005; 137: 317–327. 15618434 10.1104/pp.104.045658 PMC548862

[bib57] Garg R, Jhanwar S, Tyagi AK, Jain M. Genome-wide survey and expression analysis suggest diverse roles of glutaredoxin gene family members during development and response to various stimuli in rice. DNA Res 2010; 17: 353–367. 21044985 10.1093/dnares/dsq023 PMC2993539

[bib58] Klatt P, Lamas S. Regulation of protein function by S-glutathiolation in response to oxidative and nitrosative stress. Eur J Biochem 2000; 267: 4928–4944. 10931175 10.1046/j.1432-1327.2000.01601.x

[bib59] Lee H, Dietz KJ, Hofestädt R. Prediction of thioredoxin and glutaredoxin target proteins by identifying reversibly oxidized cysteinyl residues. J Integr Bioinform 2010; 7: 130–141. 10.2390/biecoll-jib-2010-130 20375441

[bib60] Koch K. Carbohydrate-modulated gene expression in plants. Annu Rev Plant Biol 1996; 47: 509–540. 10.1146/annurev.arplant.47.1.509 15012299

[bib61] Hoque MA, Banu MNA, Nakamura Y, Shimoishi Y, Murata Y. Proline and glycinebetaine enhance antioxidant defense and methylglyoxal detoxification systems and reduce NaCl-induced damage in cultured tobacco cells. J Plant Physiol 2008; 165: 813–824. 17920727 10.1016/j.jplph.2007.07.013

[bib62] Matysik J, Bhalu B, Mohanty P. Molecular mechanisms of quenching of reactive oxygen species by proline under stress in plants. Curr Sci 2002; 82: 525–532.

[bib63] Smirnoff N, Cumbes QJ. Hydroxyl radical scavenging activity of compatible solutes. Phytochemistry 1989; 28: 1057–1060.

[bib64] Fabro G, Kovács I, Pavet V, Szabados L, Alvarez ME. Proline accumulation and AtP5CS2 gene activation are induced by plant-pathogen incompatible interactions in *Arabidopsis*. Mol Plant Microbe Interact 2004; 17: 343–350. 15077666 10.1094/MPMI.2004.17.4.343

[bib65] Uchida A, Jagendorf AT, Hibino T, Takabe T, Takabe T. Effects of hydrogen peroxide and nitric oxide on both salt and heat stress tolerance in rice. Plant Sci 2002; 163: 515–523.

[bib66] Anchordoguy TJ, Rudolph AS, Carpenter JF, Crowe JH. Modes of interaction of cryoprotectants with membrane phospholipids during freezing. Cryobiology 1987; 24: 324–331. 3621976 10.1016/0011-2240(87)90036-8

[bib67] Shalaev EY, Steponkus PL. Phase behavior and glass transition of 1, 2-dioleoylphosphatidylethanolamine (DOPE) dehydrated in the presence of sucrose. Biochim Biophy Acta 2001; 1514: 100–116. 10.1016/s0005-2736(01)00372-8 11513808

[bib68] Couée I, Sulmon C, Gouesbet G, El Amrani A. Involvement of soluble sugars in reactive oxygen species balance and responses to oxidative stress in plants. J Exp Bot 2006; 57: 449–459. 16397003 10.1093/jxb/erj027

[bib69] Averillbates DA, Przybytkowski E. The role of glucose in cellular defenses against cytotoxicity of hydrogen peroxide in Chinese hamster ovary cells. Arch Biochem Biophys 1994; 312: 52–58. 8031146 10.1006/abbi.1994.1279

[bib70] Noctor G, Foyer CH. Ascorbate and glutathione: keeping active oxygen under control. Annu Rev Plant Biol 1998; 49: 249–279. 10.1146/annurev.arplant.49.1.249 15012235

[bib71] Meyer Y, Buchanan BB, Vignols F, Reichheld J-P. Thioredoxins and glutaredoxins: unifying elements in redox biology. Annu Rev Genet 2009; 43: 335–367. 19691428 10.1146/annurev-genet-102108-134201

[bib72] Kim SJ, Park YJ, Hwang IY, Youdim MB, Park KS, Oh YJ. Nuclear translocation of DJ-1 during oxidative stress-induced neuronal cell death. Free Radic Biol Med 2012; 53: 936–950. 22683601 10.1016/j.freeradbiomed.2012.05.035

[bib73] Dastoor Z, Dreyer JL. Potential role of nuclear translocation of glyceraldehyde-3-phosphate dehydrogenase in apoptosis and oxidative stress. J Cell Sci 2001; 114: 1643–1653. 11309196 10.1242/jcs.114.9.1643

[bib74] Li S, Lauri A, Ziemann M, Busch A, Bhave M, Zachgo S. Nuclear activity of ROXY1, a glutaredoxin interacting with TGA factors, is required for petal development in Arabidopsis thaliana. Plant Cell 2009; 21: 429–441. 19218396 10.1105/tpc.108.064477 PMC2660636

[bib75] Pautler M, Eveland AL, LaRue T et al. FASCIATED EAR4 encodes a bZIP transcription factor that regulates shoot meristem size in maize. Plant Cell 2015; 27: 104–120. 25616871 10.1105/tpc.114.132506 PMC4330574

[bib76] Yang F, Bui HT, Pautler M et al. A maize glutaredoxin gene, Abphyl2, regulates shoot meristem size and phyllotaxy. Plant Cell 2015; 27: 121–131. 25616873 10.1105/tpc.114.130393 PMC4330572

[bib77] Pujol-Carrion N, Belli G, Herrero E, Nogues A, de la Torre-Ruiz MA. Glutaredoxins Grx3 and Grx4 regulate nuclear localisation of Aft1 and the oxidative stress response in Saccharomyces cerevisiae. J Cell Sci 2006; 119: 4554–4564. 17074835 10.1242/jcs.03229

[bib78] Sharma P, Jha AB, Dubey RS, Pessarakli M. Reactive oxygen species, oxidative damage, and antioxidative defense mechanism in plants under stressful conditions. J Bot 2012. doi: 10.1155/2012/217037.

[bib79] Knight MR, Knight H. Low-temperature perception leading to gene expression and cold tolerance in higher plants. New Phytol 2012; 195: 737–751. 22816520 10.1111/j.1469-8137.2012.04239.x

[bib80] Morran S, Eini O, Pyvovarenko T et al. Improvement of stress tolerance of wheat and barley by modulation of expression of DREB/CBF factors. Plant Biotechnol J 2011; 9: 230–249. 20642740 10.1111/j.1467-7652.2010.00547.x

[bib81] Xu M, Li L, Fan Y, Wan J, Wang L. ZmCBF3 overexpression improves tolerance to abiotic stress in transgenic rice (*Oryza sativa*) without yield penalty. Plant Cell Rep 2011; 30: 1949–1957. 21811828 10.1007/s00299-011-1103-1

[bib82] Yang S, Tang XF, Ma NN, Wang LY, Meng QW. Heterology expression of the sweet pepper CBF3 gene confers elevated tolerance to chilling stress in transgenic tobacco. J Plant Physiol 2011; 168: 1804–1812. 21724293 10.1016/j.jplph.2011.05.017

[bib83] Chinnusamy V, Zhu J, Zhu JK. Cold stress regulation of gene expression in plants. Trends Plant Sci 2007; 12: 444–451. 17855156 10.1016/j.tplants.2007.07.002

[bib84] Zhu J, Shi H, Lee BH et al. An *Arabidopsis* homeodomain transcription factor gene, HOS9, mediates cold tolerance through a CBF-independent pathway. Proc Natl Acad Sci U S A 2004; 101: 9873–9878. 15205481 10.1073/pnas.0403166101 PMC470766

