# Supplementary material for: Tomato expressing Arabidopsis glutaredoxin gene AtGRXS17 confers tolerance to chilling stress via modulating cold responsive components
Source: Hortic Res. 2015 Nov 11;2:15051–. doi: 10.1038/hortres.2015.51 (PMC4641303; doi:10.1038/hortres.2015.51)
Supplement: Supplementary Figures S1–S4 and Table S1 [file hortres201551-s1.pdf]

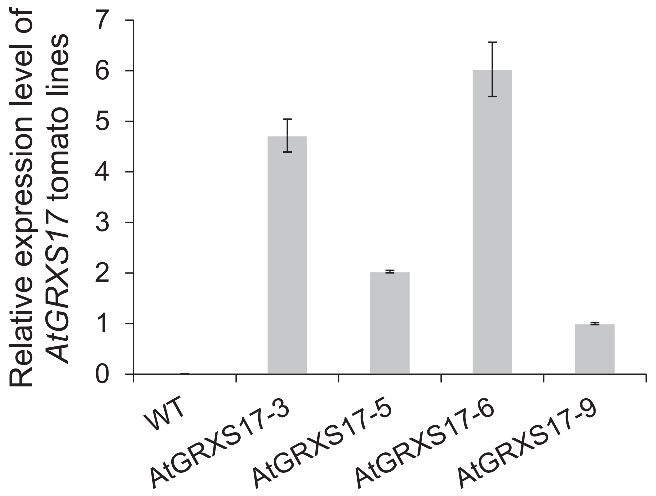

Supplementary Figure S1. qRT-PCR analysis of *AtGRXS17*-expressing T2 generation tomato plants. Relative expression level of *AtGRXS17* in four independent transgenic tomato lines (AtGRXS17-3, AtGRXS17-5, AtGRXS17-6, and AtGRXS17-9) was measured. The lowest expression level in AtGRXS17-9 was set to 1.0. Lane WT, negative control (wild-type tomato). Data represent means ± SD from three independent biological replicates.

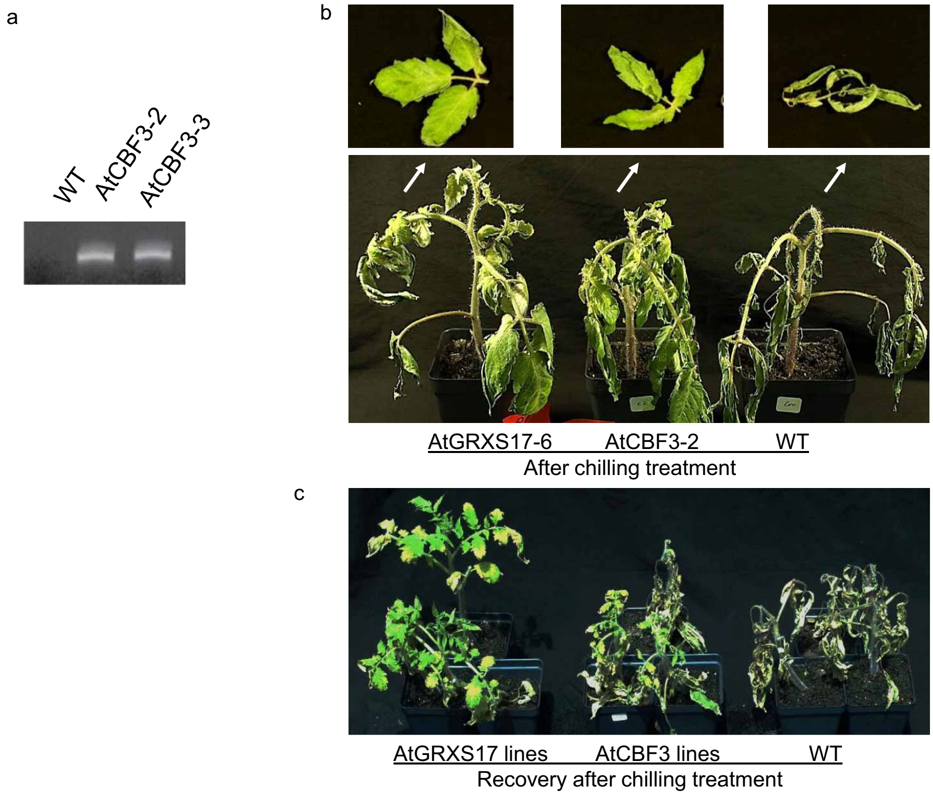

Supplementary Figure S2. Effect of ectopically expressed *AtCBF3* in tomato on chilling stress. (**a**) The integration of *AtCBF3* into tomato genome has been confirmed by PCR using HYG primers. HYG_F: 5′-GGCGACCTCGTATTGGGAATCC-3′; HYG_R: 5′-AAGTTCGACAGCGTCTCGGACC-3′. (**b**) Four-week-old *AtGRXS17*-, *AtCBF3*-expressing or wild-type plants treated under 4 ℃ (day/night) for 3 more weeks. (**C**) Two-weeks recovery after 3-week-chilling treatment. The error bars indicates means ± SD (n = 3).

© 2013 Nanjing Agricultural University

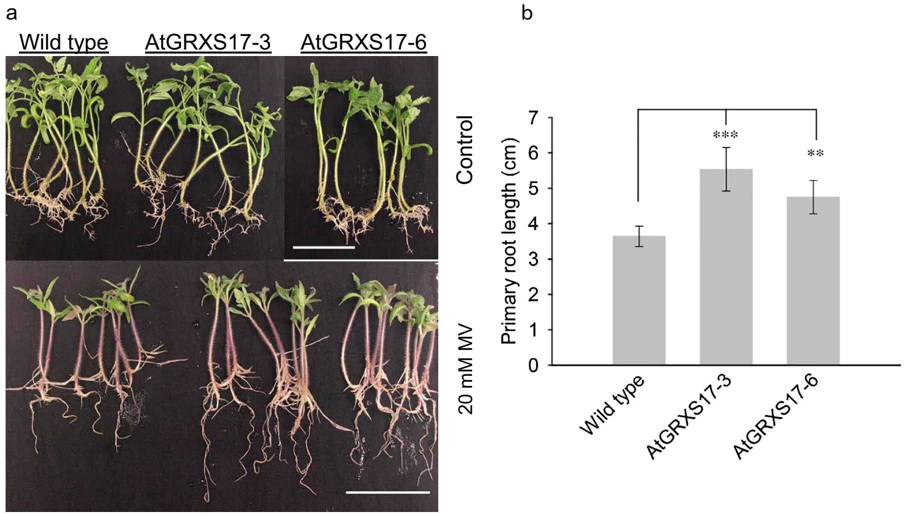

Supplementary Figure S3. Response to oxidative stress in *AtGRXS17*-expressing tomato plants. (**a**) *AtGRXS17*-expressing and wild-type tomato seedlings treated with oxidative herbicide methyl viologen (MV). Seven-day-old *AtGRXS17*-expressing and wild-type tomato seedlings were transferred onto MS media with (lower panel) or without (upper panel) 20 μM MV and incubated for 14 days. Bars = 5 cm. (**b**) Root length of wild-type and *AtGRXS17*-expressing tomato seedlings that were treated by 20 μM MV. Data represent means ± SD from eight independent biological replicates and were analyzed using Student's *t*-test. Asterisks (**,***) represent statistically significant differences between wild-type and *AtGRXS17*-expressing lines (**$P < 0.01$, ***$P < 0.001$).

© 2013 Nanjing Agricultural University

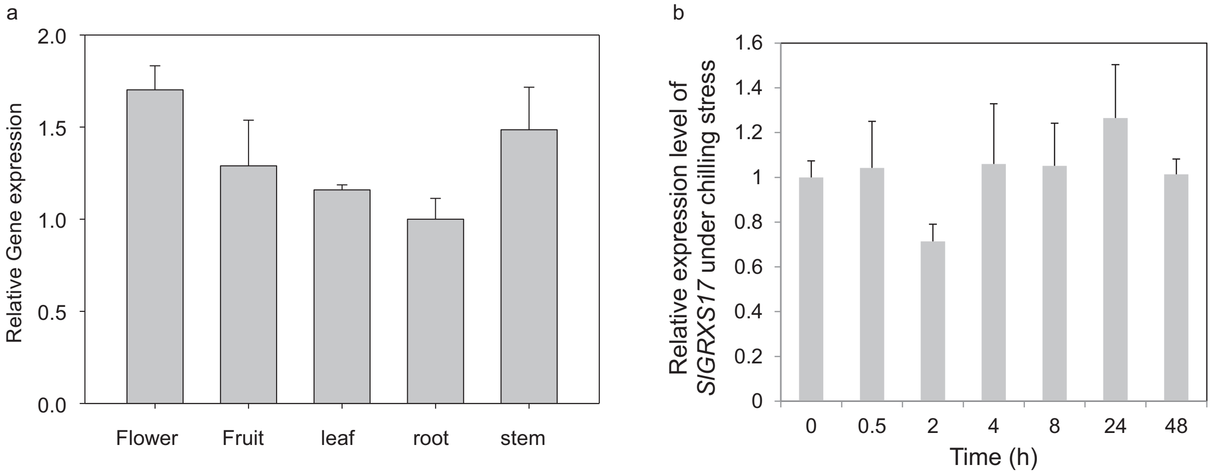

Supplementary Figure S4. qRT-PCR analysis of *SlGRXS17* expression in different tissues or organs (**a**) and under applications of chilling stress for different time periods (**b**).

© 2013 Nanjing Agricultural University

Table S1. Primers used for qRT-PCR

Gene name	Accession #	Primer sequence
SlCBF1	AY034473.1	Forward: GCTGGCAGGAAGAAGTTTCG
		Reverse: GAGTTGGAGGAAGCAGGGATAG
SlPP2Acs **(housekeeping gene)**	AY325818	Forward: CGATGTGTGATCTCCTATGGTC
		Reverse: AAGCTGATGGGCTCTAGAAATC
AtGRXS17	NM_116733	Forward: AGGTGAGTTAATTGGAGGATGT
		Reverse: TCGGATAGAGTTGCTTTGAGAT
Dehydrin Ci7	AK224734	Forward: GAACCCAAGGAGGAGGAAA
		Reverse: TCTTCTTCTTGATCTTCTGTCCAT
Dehydrin-like	AI775935	Forward: CATATCAATAAGCGGAGGAGAG
		Reverse: GAAAGCAGTTAAACACAAGATAGA
Proteinase inhibitor	BT012682	Forward: ATCGTTTCAAGGGACCAT
		Reverse: AATCCACGGCAATTACCA
Glycine rich	NM_001247128	Forward: CTACTTCTGAAGAGGATTCCAAGA
		Reverse: CACCACCACCACCACTAC
SlCAT1	NM_001247898	Forward: ATTGCTGCTGGAAACTATCCTGAG
		Reverse: GGTCCAATACGGTGTCTCTGAGTA
SlSOD	NM_001247840	Forward: CGGTGTGGTTGGTTTGAC
		Reverse: AAAGTGTTGGCAAGTGTGTA
SlFESOD	NM_001246860	Forward: GCATACAAACCTGAAGACAAA
		Reverse: TGACACCAACTTCTCCATAA
SlTPX1	L13654	Forward: GGTCTGTTCCAATCCGATG
		Reverse: CACAATGCTTCCTGATTTCAC
SlTPX2	NM_001247715	Forward: CCATCAATGACAACACCACAA
		Reverse: CGGTTCGGCATAGAATTGTT
